# The Decrease in Mitochondrial DNA Mutation Load Parallels Visual Recovery in a Leber Hereditary Optic Neuropathy Patient

**DOI:** 10.3389/fnins.2018.00061

**Published:** 2018-02-09

**Authors:** Sonia Emperador, Mariona Vidal, Carmen Hernández-Ainsa, Cristina Ruiz-Ruiz, Daniel Woods, Ana Morales-Becerra, Jorge Arruga, Rafael Artuch, Ester López-Gallardo, M. Pilar Bayona-Bafaluy, Julio Montoya, Eduardo Ruiz-Pesini

**Affiliations:** ^1^Departamento de Bioquímica, Biología Molecular y Celular, Universidad de Zaragoza, Zaragoza, Spain; ^2^Instituto de Investigación Sanitaria de Aragón (IIS Aragón), Zaragoza, Spain; ^3^Centro de Investigaciones Biomédicas En Red de Enfermedades Raras (CIBERER), Barcelona, Spain; ^4^Servicio de Oftalmología Pediátrica, Hospital Sant Joan de Déu, Barcelona, Spain; ^5^Servicio de Oftalmología, Hospital Universitario de Bellvitge, L'Hospitalet de Llobregat, Barcelona, Spain; ^6^Servicio de Bioquímica, Hospital Institut de Recerca Sant Joan de Déu, Barcelona, Spain; ^7^Fundación ARAID, Zaragoza, Spain

**Keywords:** LHON, childhood-onset disease, visual recovery, mtDNA, heteroplasmic mutation

## Abstract

The onset of Leber hereditary optic neuropathy is relatively rare in childhood and, interestingly, the rate of spontaneous visual recovery is very high in this group of patients. Here, we report a child harboring a rare pathological mitochondrial DNA mutation, present in heteroplasmy, associated with the disease. A patient follow-up showed a rapid recovery of the vision accompanied by a decrease of the percentage of mutated mtDNA. A retrospective study on the age of recovery of all childhood-onset Leber hereditary optic neuropathy patients reported in the literature suggested that this process was probably related with pubertal changes.

## Introduction

Leber hereditary optic neuropathy (LHON) is a type of blindness, usually characterized by severe central vision loss in one eye soon followed by the fellow eye, associated dense scotomas and impaired color vision. Ninety percent of the patients with this disease have been associated with one of the following point mutations within the mitochondrial DNA (mtDNA): m.3460G>A, m.11778G>A, and m.14484T>C. A plethora of rare mutations is responsible for the remaining 10% and some of them have been previously associated to other mitochondrial phenotypes.

LHON mainly affects young adult men. The peak age of onset is in the second and third decades of life (Majander et al., 2017). The onset of the disease in childhood is relatively rare. Thus, it has been reported that <10% of patients were 12 year-old or younger at the time of diagnosis (Majander et al., 2017). Notably, in this patient population, the rate of spontaneous visual recovery is very high (Majander et al., 2017). However, the reason for such spontaneous recovery is unknown and there is hardly any information on either biochemical nor lifestyle of the patients around the recovery period.

Hereby in the present paper, we report a case of a child suffering LHON who presents a rare mtDNA mutation. Biochemical, cellular and genetic studies performed on patient' fibroblasts and cybrids show that this mutation is pathological. Interestingly enough, the patient rapidly recovered his vision. We also discuss a potential correlation of the age of recovery of childhood-onset LHON patients with pubertal changes since two thirds of these patients recovered sight before the age of 13.

## Materials and methods

### Case report

**Figure 1:**
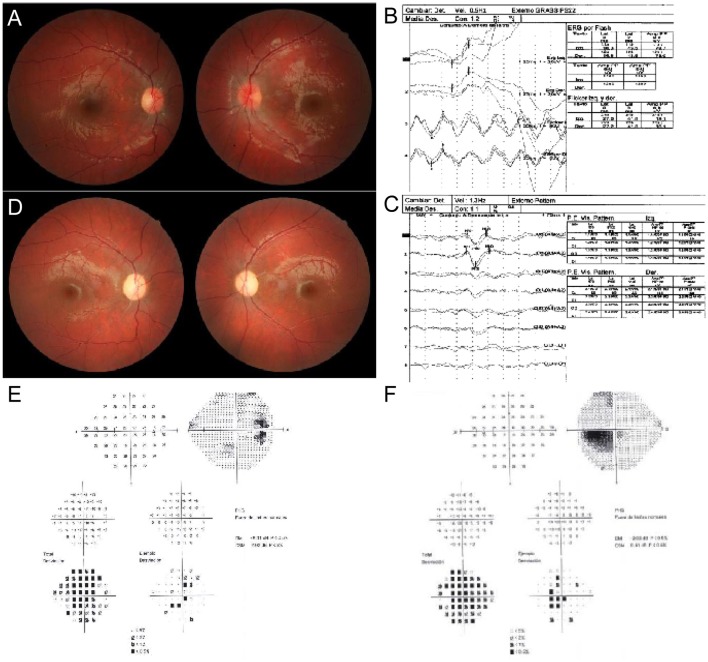
Ocular exam. **(A)** Fundus photography. RE shows temporal optic disc pallor. LE shows a congestive disc with telangiectasies. **(B)** Electroretinogram. **(C)** Visual evoked potential pattern. Bilateral delay of conduction through the visual pathways is observed. **(D)** Fundus photography. Bilateral pallor of the optic discs remains. **(E,F)** Visual fields analysis. RE and LE show a paracentral and a cecocentral scotoma, respectively.

A 10 year-old male came to our department referring bilateral and painless visual loss. Upon examination, visual acuity (VA) was 20/400 (1.3 logarithm of the minimum angle resolution- LogMAR) right eye (RE) and 20/100 (0.7 LogMAR) left eye (LE), Ishihara Test: 0/20 RE and 5/20 LE. Pupils were slightly anisocoric (RE > LE), with normal responses to light. Anterior segment was normal in both eyes, and ocular fundus showed temporal optic disc pallor RE and a congestive disc with telangiectasies LE (Figure 1A). Brain magnetic resonance was performed with normal results. The electroretinogram study was normal and visual evoked potential study showed a bilateral delay of conduction through the visual pathways suggestive of bilateral optic neuropathy (Figures 1B,C). After 6 months the RE showed a favorable evolution, with VA of 20/25 (0.1 LogMAR) but VA in the LE stayed at 20/100 (0.7 LogMAR). Ishihara test was 18/20 in RE and 1/20 in LE, and a slight relative afferent pupillary defect was observed in the LE. The visual fields showed a paracentral scotoma in the RE and a cecocentral scotoma in the LE. Fundoscopy revealed bilateral marked optic disc pallor (LE > RE) with an associate vascular attenuation (Figure 1D). There was a stabilization of the clinical picture over the following 2 years. Ultimately, the patient has experienced a favorable evolution after 4 years from the beginning of the clinical symptoms. His VA has presently improved to 20/20 RE (0.0 LogMAR) and 20/40 LE (0.3 LogMAR) with an improvement of the Ishihara test to 20/20 RE and 20/20 LE. Visual fields show minimal defects in the RE and a 5 degree central scotoma in the LE. However, bilateral pallor of the optic discs remains in the ocular fundus (Figures 1E,F). This study was approved by and carried out in accordance to the recommendations of Institutional Review Board from the Government of Aragón (CEICA CP-12/2014). The patient's mother written informed consent was obtained for their participation in the study, in accordance with the Declaration of Helsinki, and for publication of the case report.

### Molecular-genetic analyses

Total DNA was extracted by standard methods. Screening for the three primary LHON mutations was performed by polymerase chain reaction (PCR)/restriction fragment length polymorphism (RFLP) (Supplementary Table 1). The complete mtDNA was amplified and sequenced according to previously described protocols (Supplementary Table 2; Gomez-Duran et al., 2010). The percentage of m.13094T>C transition was analyzed by PCR/RFLP by using protocols described elsewhere (Valente et al., 2009). The mtDNA content was measured, in triplicate in three independent experiments, by the real-time quantitative reverse transcription-PCR (RT-qPCR) method using an Applied Biosystems StepOne™ Real-Time PCR System Thermal Cycling Block, as described elsewhere (Andreu et al., 2009).

### Production of transmitochondrial cell lines and cell culture

To homogenize nuclear and environmental factors, we produced transmitochondrial cell lines (cytoplasmic hybrids or cybrids) with the osteosarcoma 143B rho^0^ nuclear background using patient and control platelets (Chomyn, 1996). These cybrids, as well as patient and control fibroblasts, were grown in Dulbecco's modified eagle medium with no antibiotics and containing glucose (4.5 g/l), pyruvate (0.11 g/l) and 5 or 10% of fetal bovine serum (FBS), respectively.

### Biochemical investigations

The analyses of oxygen consumption, ATP, mitochondrial inner membrane potential (MIMP) and H_2_O_2_ levels were performed in triplicate in 3 independent experiments according to previously described protocols (Gomez-Duran et al., 2010; Llobet et al., 2015). The activity of several mitochondrial complexes was analyzed using BN-PAGE *in-gel* activity technique (Wittig et al., 2007). Western blots in cellular lysates were performed using Total OXPHOS Human WB Antibody Cocktail (1:1,000, ab110411, ABCAM), anti-LC3B (1:1,000, L7543, Sigma) and anti-actin (1:2,000, A2066, Sigma) as primary antibodies (Lopez-Gallardo et al., 2009).

### Molecular modeling

The three-dimensional structure of the bovine p.MT-ND5 (PDB 5LNK) was obtained with the RasMol 2.6 program (http://www.rasmol.org).

### Statistical analysis

The statistical package StatView 6.0 was used to perform all the statistics. Data for mean and standard deviation are presented. The unpaired two-tailed *t*-test was used to compare parameters. *P*-values lower than 0.05 were considered statistically significant.

## Results

**Figure 2:**
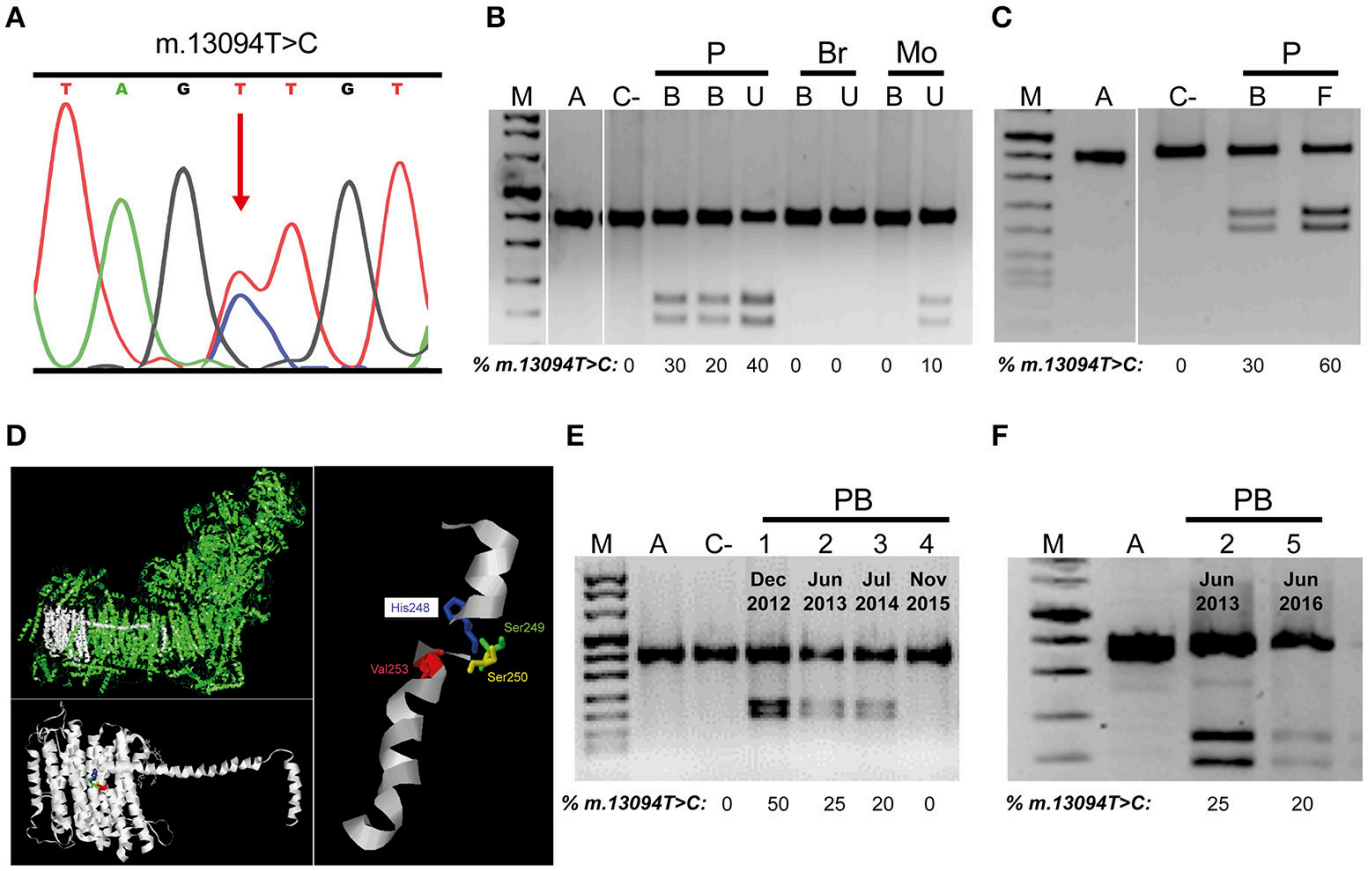
mtDNA m.13094T>C mutation. **(A)** Electropherogram showing the presence of the heteroplasmic mutation in the patient. **(B,C)** Mutation load in different members of the family. **(D)** Molecular model of p.MT-ND5 subunit. Respiratory complex I (CI), p.MT-ND5 polypeptide and transmembrane helix 8 (TMH8) showing the affected amino acid. **(E,F)** Mutation load variation through time in patient's blood. M, DNA molecular weight marker VIII (Roche); A, amplicon; C-, negative control; P, patient; Br, brother; Mo, mother; B, U, F, patient blood, urine and fibroblasts; PB, 1-5, patient blood indicating the dates. Uncropped images for gels in **B,C** can be found in Supplementary Figure 2A,B, respectively.

The three most common LHON mutations (m.3460G>A, m.11778G>A, and m.14484T>C) are responsible for approximately 90% of all LHON cases (Majander et al., 2017). Therefore, we tested their presence by PCR-RFLP and ruled them out. Next, we sequenced the whole mtDNA from blood cells and assigned it to mitochondrial haplogroup H1 (Van Oven and Kayser, 2009). This mtDNA harbored 4 private mutations (GenBank MG386502): m.11113T>C synonymous and homoplasmic mutation in the *MT-ND4* gene; m.13094T>C in the *MT-ND5* gene; m.15527C>T in the *MT-CYB* gene; and the highly frequent and homoplasmic m.16295C>T mutation in the *MT-CR* control region. One of the two non-synonymous variants, the m.15527C>T transition, is a homoplasmic mutation (Supplementary Figure 1A) which has not been reported in 37,545 published human mtDNA sequences (GenBank, November 3, 2017), although it was present in homoplasmy in mother's blood and urine (Supplementary Figure 1B). This mutation causes a proline to serine substitution in p.MT-CYB position 261. This proline is conserved in 94.7% of 4,988 eukaryotic (from protists to mammals) p.MT-CYB sequences (Martin-Navarro et al., 2017). Pathogenicity predictors, such as MutPred (Pereira et al., 2011), PolyPhen-2 and Mitoclass.1 (Martin-Navarro et al., 2017), consider this amino acid substitution as a pathogenic mutation. The m.13094T>C mutation has not been reported in these 37,545 human sequences. This is a heteroplasmic mutation (Figure 2A) and its percentage varies between patient tissues (Figures 2B,C). Said mutation was not found in blood or urine of his younger brother nor in his mother's blood but it was present, in a low percentage (10%), in the urine of his mother (Figure 2B). This m.13094T>C transition provokes a valine to alanine change in p.MT-ND5 position 253 (Figure 2D). The valine is conserved in 99.7% of 5,159 eukaryotic p.MT-ND5 sequences (Martin-Navarro et al., 2017). This Val253 is located in the p.MT-ND5 transmembrane helix 8 (TMH8), following a serine pair (Ser249 and Ser250) that distorts TMH8 (Zhu et al., 2016), and a key His248 sit on a flexible loop of the discontinuous TMH8. This mid-membrane break/loop lend flexibility to key protonable residues (Fiedorczuk et al., 2016; Figure 2D). PolyPhen-2 and Mitoclass.1 consider this amino acid substitution as a pathogenic one, but as for MutPred its pathogenicity is low.

Along 3 years, four different blood analysis were performed and the percentage of m.13094T>C mutation decreased from 50% to undetectable (Figure 2E), but the m.15527C>T transition stayed homoplasmic (Supplementary Figure 1C). Simultaneously, there was an improvement in visual acuities to an eventually almost complete visual recovery. However, another blood sample, 8 months later, showed a 15–20% of the m.13094T>C mutation (Figure 2F), although the vision was not altered. mtDNA copy numbers in blood from different ages [Patient Blood 2 (PB2), 78.8 copies/cell ± 4.71 (2); PB3, 35.0 copies/cell ± 0.22 (2); PB4, 55.5 copies/cell ± 2.75 (2); PB5, 69.3 copies/cell ± 0.30 (2)] were not correlated with the percentage of mutation. As LHON affected cells are the retinal ganglion cells (RGCs), this visual recovery with reduction in blood mutation load was probably accompanied by a decline in the RGCs mutation load. The onset of LHON is relatively rare in childhood, but their prognosis is more favorable (Majander et al., 2017). Thus, from 14 childhood-onset LHON patients (age of onset, 2–12 years) who showed visual recovery (exactly dated), 10 have got it before the age of 13 (Mackey and Howell, 1992; Kawasaki and Borruat, 2005; Sharkawi et al., 2012; Majander et al., 2017).

**Figure 3:**
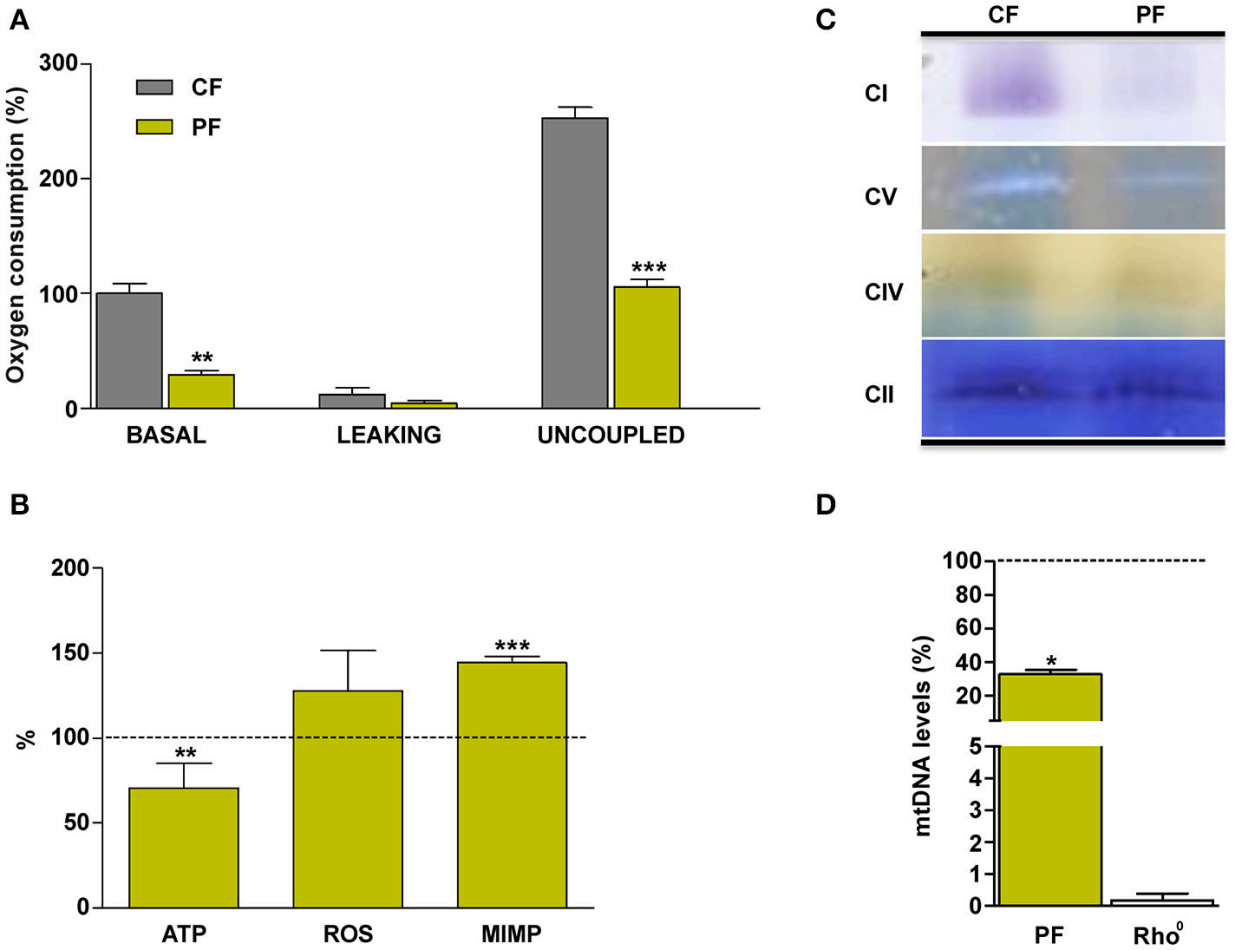
Mitochondrial biochemical characterization of the patient's fibroblasts. **(A)** Oxygen consumption. **(B)** ATP and reactive oxygen species (ROS) levels and mitochondrial inner membrane potential (MIMP). **(C)** *In gel* complexes activity. **(D)** mtDNA quantity. CF, PF, CI, CII, CIV, CV, and Rho^0^ code for control fibroblasts, patient fibroblasts, complex I, complex II, complex IV, complex V, and cells without mtDNA, respectively. Dashed line represents the control fibroblast values. ^*^, ^**^, ^***^ indicate < 0.05, 0.01, and 0.001 *p*-values, respectively.

Next, we checked cellular effects of these mtDNA mutations in the patient's fibroblasts. Basal and uncoupled respiration and ATP levels were significantly decreased in patient's vs. control's fibroblasts (Figures 3A,B). There was no difference in the levels of hydrogen peroxide (Figure 3B). On the other hand, mitochondrial inner membrane potential was significantly increased in patient's fibroblasts (Figure 3B). An *in gel* activity analysis showed an important decrease in complex I (CI) and V (CV) activities (Figure 3C). The mtDNA amount was also significantly lower in the patient's fibroblasts (Figure 3D).

**Figure 4:**
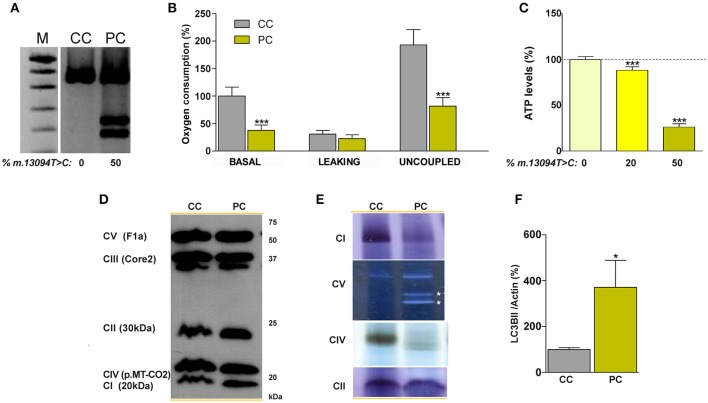
Biochemical characterization of the normal and 50% mutation cybrid cell lines. **(A)** Gel showing the percentage of the m.13094T>C mutation. **(B)** oxygen consumption. **(C)** ATP levels in cybrids harboring different (0, 20, and 50%) m.13094T>C mutation load. **(D)** Levels of different OXPHOS subunits (in brackets) in cellular lysates. **(E)** *In gel* complexes activity. White asterisks denote CV subcomplexes. **(F)** Western blot quantification of the LC3BII autophagy marker, normalized to actin, in cellular lysates. M, DNA molecular weight marker VIII (Roche); CC and PC denote control and patient (50% mutation) cybrids, respectively. CI, CII, CIII, CIV, and CV code for complex I, complex II, complex III, complex IV and complex V, respectively. Dashed line represents the control cybrid values. ^*^, ^***^ indicate < 0.05 and 0.001 *p*-values, respectively. Uncropped image for gel in **(A)** can be found in Supplementary Figure 2C. Results for **(F)** can be found in Supplementary Figure 2D.

In the patient's vs control's fibroblasts comparison, culture conditions are homogenized, but nuclear DNA (nDNA) and mtDNA genetic backgrounds differ. On the contrary, in cybrids the nDNA is also homogeneous and they only differ in the mtDNA genotype. Therefore, we used osteosarcoma 143B rho^0^ cells to build mutant and control cybrids (Figure 4A and Supplementary Figure 1D). The short terminal repeats (STR) markers reported in the American Type Culture Collection (ATCC) for the osteosarcoma 143B cell line did not differ from those of the cell lines used in this work, and they are the same than those previously reported in other osteosarcoma 143B cybrids (Lopez-Gallardo et al., 2014), thus confirming the nDNA homogeneity. Basal and uncoupled oxygen consumptions were significantly lower in mutant cells (Figure 4B), and this was accompanied by lesser cell ATP amount. The ATP levels were higher in cybrids with lower percentages of m.13094T>C mutation, although they were homoplasmic for the m.15527C>T transition (Figure 4C and Supplementary Figure 1D). Surprisingly, we found an inverse relationship between the percentage of m.13094T>C mutation and the mtDNA copy numbers [0%, 950 copies/cell ± 1 (1); 20%, 737 copies/cell ± 159 (8); 50%, 541 copies/cell ± 41 (3)]. Although levels of nDNA (CI-20kDa, CII-30kDa, CIII-Core2, and CV-F1a) and mtDNA (CIV-p.MT-CO2) subunits did not differ (Figure 4D), the *in gel* activity analysis showed an important decrease in CI and CIV activities, and CV sub-complexes were also observed (Figure 4E). Autophagy, determined as LC3B-II/Actin levels, was significantly increased in mutant cybrids (Figure 4F).

## Discussion

We found two potential mtDNA pathologic mutations in the patient. The change in ATP levels of mutant cybrids, despite all of them harboring a homoplasmic m.15527C>T transition, along with other considerations make us rule out this mutation as the pathologic one for this patient. However, the m.13094T>C heteroplasmic mutation had been previously associated to two other patients. The first was a 7 year-old child suffering from Ataxia and Progressive External Ophthalmoplegia (PEO). The skeletal muscle biopsy was morphologically normal. Muscle and fibroblast were also biochemically normal. However, mutant fibroblasts had a clear reduction in CI *in gel* activity and CI-sub-complexes in a 2D-BNGE WB analysis. In osteosarcoma 143B cybrids, the CI/CS ratio negatively correlated with the percentage of mutation and the 80% mutant cybrid showed CI sub-complexes. Mutant and control CI amount was comparable in fibroblasts and also in cybrids (Valente et al., 2009). This heteroplasmic mutation was also found in a 34 year-old woman with Kearns-Sayre syndrome (Lax et al., 2012a although, in Lax et al., 2012b, this patient is referred as suffering from mitochondrial encephalomyopathy with lactic acidosis and stroke-like episodes (MELAS)/Leigh syndrome (LS), myoclonus and fatigue). A vascular smooth muscle cell loss was observed along with thinning of the vascular smooth muscle cell layer and 50% of neurons were lost from the olivo-cerebelum (Lax et al., 2012a,b). On the other hand, besides m.13094T>C, several p.MT-ND5 mutations have been also associated with LHON or LHON-like phenotypes, and some of them were previously associated to other phenotypes, such as MELAS or LS (www.mitomap.org). Our evidences, along with those from these two other articles, strongly support the m.13094T>C transition as a pathologic mutation for PEO, MELAS/LS (KSS), or LHON. Probably, tissue distribution and percentage of the mutation are responsible for these different phenotypes. Interestingly, this mutation seemed to trigger a decrease in mtDNA amount that could be responsible for the OXPHOS multienzymatic deficit in patient fibroblasts and cybrids.

The decrease in mutation levels is probably responsible for the patient's recovery. A reduction in the mutation load in proliferative tissues has been observed in some longitudinal studies for LHON mutations (Howell et al., 2000; Jacobi et al., 2001; Puomila et al., 2002; Kaplanova et al., 2004). However, the decrease is mostly moderate. Only 2 patients and 2 carriers harboring the m.3460G>A LHON mutation showed a reduction ≥10% (Supplementary Table 3). One of the patients, who recovered the vision, showed a reduction in the mutation load from 46 to 35% over the following 5 years. Our patient, who also recovered vision, showed a 50% decrease in the blood mutation load. Blood is not the affected tissue in LHON patients, but RGCs is. These cells are mostly inaccessible for genetic tests. Then, why should the blood mutation load be related to the visual health? Despite tissue-specific directional selection for different mtDNA genotypes has been reported (Jenuth et al., 1997), it was also observed that a high amount of mutated mtDNA in leukocytes was also correlated to a high proportion in other tissues (Juvonen et al., 1997).

We have not been able to note any particular changes in the patient's life style as a potential cause of this decrease in the mutation load and vision recovery. We had previously found higher mtDNA content in peripheral blood cells of unaffected heteroplasmic mutation carriers with respect to the affected ones (Bianco et al., 2017). Moreover, many patients showing visual recovery are homoplasmic individuals. In these cases, their improvement cannot be associated to a reduction in the mutation load. Visual recovery of these individuals may also be accompanied by an increase in their RGCs mtDNA levels mirrored in their blood mtDNA levels. In fact, mtDNA content in peripheral blood cells is higher in unaffected homoplasmic mutation carriers with respect to the affected ones (Bianco et al., 2016). Remarkably, more than two thirds of unaffected homoplasmic carriers are female (Bianco et al., 2016), and it has been observed that oestradiol increases mtDNA content, which it could explain the lower LHON prevalence in females (Giordano et al., 2011). LHON is also less prevalent in prepubertal girls (Majander et al., 2017). Interestingly, oestradiol values are higher in prepubertal girls than in prepubertal boys and these levels increase with age and pubertal stage in both sexes (Ikegami et al., 2001; Janfaza et al., 2006). This rise in prepubertal oestradiol levels could be responsible for the high spontaneous visual recovery rate of childhood LHON patients (Majander et al., 2017). In this case, prepubertal oestradiol concentration might be a biomarker for childhood LHON. More importantly, transiently increasing oestradiol concentrations perhaps avoided the blindness or accelerated the recovery.

## Author contributions

SE, CH-A, CR-R, DW, EL-G, and MB-B performed research, designed experiments, collected and analyzed data, revised paper; MV, AM-B, JA, and RA collected and analyzed data and revised paper; JM and ER-P directed the project, designed experiments, analyzed data, wrote and revised paper.

### Conflict of interest statement

The authors declare that the research was conducted in the absence of any commercial or financial relationships that could be construed as a potential conflict of interest.
