# Supplementary material for: The Decrease in Mitochondrial DNA Mutation Load Parallels Visual Recovery in a Leber Hereditary Optic Neuropathy Patient
Source: Front Neurosci. 2018 Feb 9;12:61. doi: 10.3389/fnins.2018.00061 (PMC5811516; doi:10.3389/fnins.2018.00061)
Supplement: Supplementary file 1 [file Presentation1.PDF]

Supplementary Material

The Decrease in Mitochondrial DNA Mutation Load Parallels Visual Recovery in a Leber Hereditary Optic Neuropathy Patient

Sonia Emperador, Mariona Vidal, Carmen Hernández-Ainsa, Cristina Ruiz-Ruiz, Daniel Woods, Ana Morales-Becerra, Jorge Arruga, Rafael Artuch, Ester López-Gallardo, M.Pilar Bayona-Bafaluy, Julio Montoya*, Eduardo Ruiz-Pesini*

* Correspondence: Eduardo Ruiz-Pesini (eduruiz@unizar.es) and Julio Montoya (jmontoya@unizar.es).

1. Supplementary Tables and Figures

Supplementary Tables

Supplementary Table 1. Protocol for the analysis of most frequent LHON mutations.

MUTATION	PRIMER	SEQUENCE 5' --> 3'	5´-NUCLEOTIDE POSITION	SIZE (bp)	Tm	RESTRICTION ENZIME	RFLP PATTERNS	
							NORMAL	MUTANT
m.11778G>A	L1L	TACTCTTCAATCAGCCACAT	11621	307	60 ºC	MaeIII	255, 29, 23	131, 124, 29, 23
	L1H	TTGATCAGGAGAACGTGGTT	11927					
m.3460G>A	L3L	TCGCAATGGCATTCCTAATG	3350	331	66 ºC	Hsp92I	222, 109	331
	L3H	GAGTTTGATGCTCACCCTGA	3680					
m.14484T>C	L5L	CTCAATAGCCATCGCTGTAGT ATATCCAAAGACATGCA*	14446	135	66 ºC	HphI	135	108, 27
	L5H	TTGATTGTTAGCGGTGTGGT	14580					

*L5L primer introduces a "mispairing" at m.14481C>T nucleotide position.

PCR protocol: 94 ºC 5 min / (94 ºC 45 s / Tm 30 s / 72 ºC 2 min) 35 cycles / 72 ºC 5 min

Supplementary Table 2. Protocol for whole mtDNA sequencing.

PCR					SEQUENCING	
**AMPLICON**	**PRIMER**	**SEQUENCE 5' --> 3'**	**SIZE (bp)**	**Tm**	**PRIMER**	**SEQUENCE 5' --> 3'**
**F1**	hmtL 569	AACCAAACCCCAAAGACACC	2452	66 ºC	hmtL 569	AACCAAACCCCAAAGACACC
	hmtH 2982	CTGATCCAACATCGAGGTCG			hmtL 1305	GTAAGCGCAAGTACCCACG
					hmtL 2070	AATTTGCCCACAGAACCCTC
**F2**	hmtL 2797	GTCCTAAACTACCAAACCTGC	2417	65 ºC	hmtL 2797	GTCCTAAACTACCAAACCTGC
	hmtH 5174	GTGTTAGTCATGTTAGCTTG			hmtL 3568	CGCTCTTCTACTATGAACCC
					hmtL 4322	ATAATAGGAGCTTAAACCCCC
**F3**	hmtL 5061	AGCAGTTCTACCGTACAACC	2476	68 ºC	hmtL 5061	AGCAGTTCTACCGTACAACC
	hmtH 7497	TTTGAAAAAGTCATGGAGGCC			hmtL 5828	GAAAATCACCTCGGAGCTGG
					hmtL 6563	ACCTCAACACCACCTTCTTC
**F4**	hmtL 7336	GATTTGAGAAGCCTTCGCTTC	2524	66 ºC	hmtL 7336	GATTTGAGAAGCCTTCGCTTC
	hmtH 9819	GCTATAGGGTAAATACGGGC			COIIL 8018	CCGATTGAAGCCCCCATTCG
					hmtL 8815	CTCATTTACACCAACCACCC
**F5**	hmtL 9611	TCCCACTCCTAAACACATCC	2539	66 ºC	hmtL 9611	TCCCACTCCTAAACACATCC
	hmtH 12111	AAACCCGGTAATGATGTCGG			hmtL 10380	TCTGGCCTATGAGTGACTAC
					hmtL 11107	TTCACAGCCACAGAACTAATC
**F6**	L1L	TACTCTTCAATCAGCCACAT	2977	67 ºC	L1L	TACTCTTCAATCAGCCACAT
	hmtH 14559	GATTGTTAGCGGTGTGGTCG			hmtL 12640	TCGTTACATGGTCCATCATAG
					hmtL 13389	TCCATCATCCACAACCTTAAC
**F7**	hmtL 14130	TCTTCCCACTCATCCTAACC	2590	66 ºC	hmtL 14130	TCTTCCCACTCATCCTAACC
	hmtH 112	ACAGATACTGCGACATAGGG			hmtL 14838	TCCAACATCTCCGCATGATG
					hmtL 15591	TTCGCCTACACAATTCTCCG
**F8**	hmtL 15591	TTCGCCTACACAATTCTCCG	1643	66 ºC	hmtL 16365	GTCAAATCCCTTCTCGTCCC
	hmtH 626	TTTATGGGGTGATGTGAGCC			hmtH 626	TTTATGGGGTGATGTGAGCC

PCR protocol: 94 ºC 5 min / (94 ºC 45 s / Tm 30 s / 72 ºC 3 min) 35 cycles / 72 ºC 5 min

Supplementary Table 3. Change in LHON mutation load along the time.

Individual	Blood 1	Blood 2	Difference	Interval	Reference
II-4	9.7 (4.7)	2.9 (1.7)	6.8 (5.7)	5½ (5½)	Howell et al., 2000
III-1	36.1	28.5	7.6	6¼	Howell et al., 2000
III-2	7.3	5.0	2.3	6¼	Howell et al., 2000
IV-2	32.3 (33.0)	26.1 (26.9)	6.2 (6.1)	5½ (6¼)	Howell et al., 2000
IV-4	41.1	30.9	10.2	5½	Howell et al., 2000
II-2	46.0 (47.0)	35.0 (31.0)	11.0 (16.0)	5⅔	Jacobi et al., 2001
II-3	30.0 (32.0)	18.0 (22.0)	12.0 (10.0)	5⅔	Jacobi et al., 2001
II-4	42.0	40.0	2.0	5⅔	Jacobi et al., 2001
II-2	57.8	56.0	1.8	4	Puomila et al., 2002
II-3	23.7	22.0	1.7	5	Puomila et al., 2002
III-1	21.1	21.0	0.1	5	Puomila et al., 2002
III-2	47.3	42.0	5.3	12	Puomila et al., 2002
I-2	30.0	23.9	6.1	1½-3?	Kaplanova et al., 2004
II-5	16.4	10.8	5.6	1½-3?	Kaplanova et al., 2004
II-6	33.8	30.9	2.9	1½-3?	Kaplanova et al., 2004
II-8	79.1	66.3	12.8	4	Kaplanova et al., 2004
II-10	80.6	76.6	4.0	1½-3?	Kaplanova et al., 2004
I-4	50.7	49.6	1.1	1½-3?	Kaplanova et al., 2004
II-1	50.0	0	50.0	2⅚	This article

Initial heteroplasmy < 98 % and > 2 %. Black, red and blue colors code for m.3460G>A, m.11778G>A, and m.13094T>C mutations, respectively. Grey background indicates LHON patient.

Supplementary Figures

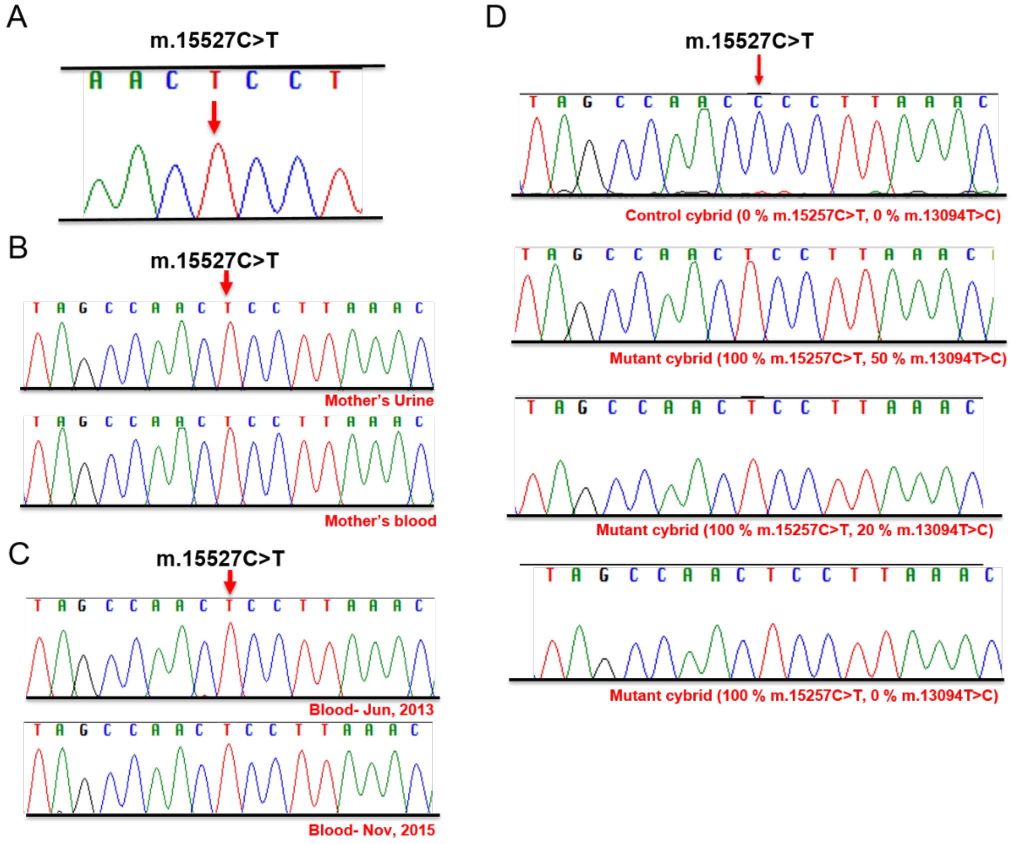

Supplementary Figure 1. Electropherograms of m.15527C>T mutation. A) Patient's blood. B) Mother's samples. C) Mutation load variation along the time. D) Cybrids.

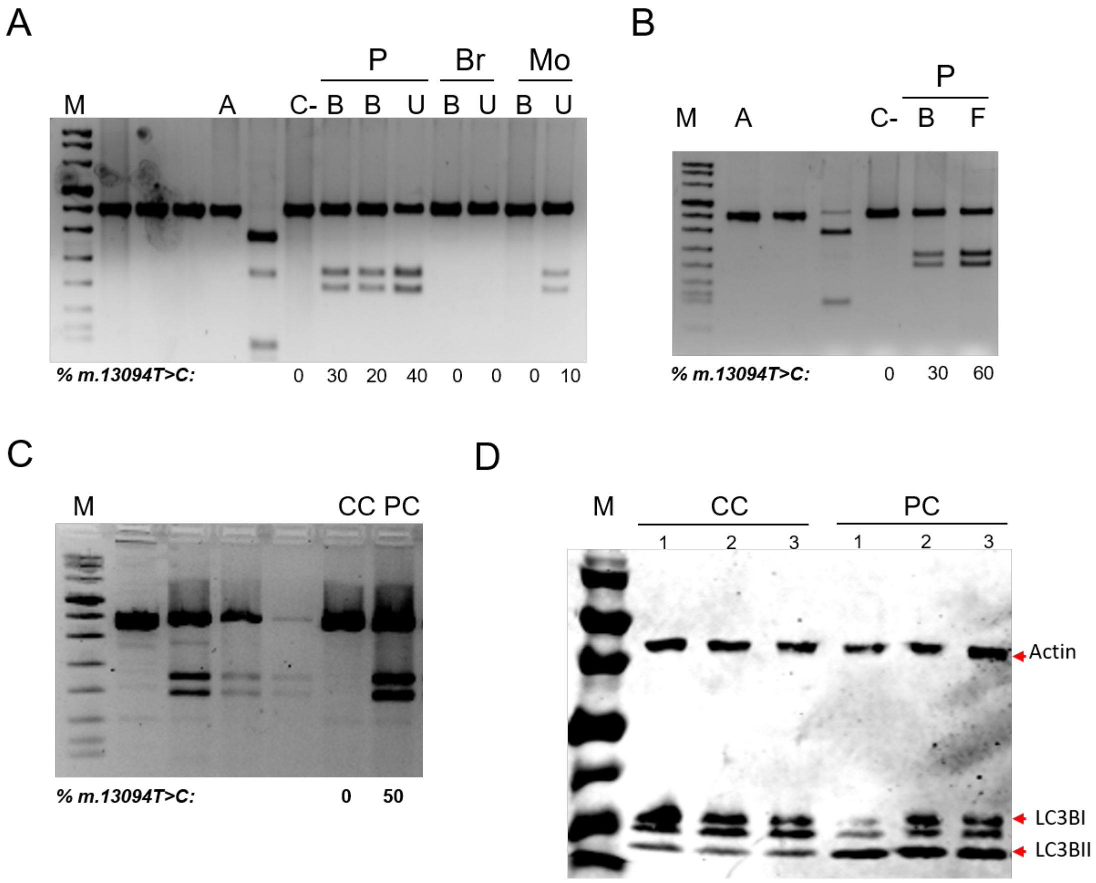

Supplementary Figure 2. Uncropped images for gels from Figures 2B (Figure S2A), 2C (Figure S2B), 4A (Figure S2C) and 4F (Figure S2D). M, DNA molecular weight marker VIII (Roche); A, amplicon; C-, negative control; P, patient; Br, brother; Mo, mother; B, blood; U, urine; F, fibroblasts; CC, control cybrid; and PC, patient cybrid, respectively.
